# Silicon Dioxide Multi-Mode Interference Spectrometers

**DOI:** 10.3390/mi17040453

**Published:** 2026-04-07

**Authors:** James G. Harkness, Denghui Pan, Helio Ramollari, Thomas D. Yuzvinsky, Holger Schmidt, Aaron R. Hawkins

**Affiliations:** 1 Department of Electrical and Computer Engineering, Brigham Young University, Provo, UT 84602, USA; 2 Department of Electrical and Computer Engineering, University of California Santa Cruz, Santa Cruz, CA 95064, USA; dpan13@ucsc.edu (D.P.); hramolla@ucsc.edu (H.R.); yuzvinsky@ucsc.edu (T.D.Y.); hschmidt@ucsc.edu (H.S.)

**Keywords:** reconstructive spectrometer, SU-8, PECVD silicon dioxide, surface roughening, micro-spectrometer, multi-mode interferometer

## Abstract

A multi-mode interferometer (MMI) spectrometer is a type of reconstructive micro-spectrometer based on imaging light propagation patterns in MMI waveguides. A waveguide scattering surface accentuates imaging light patterns in the multi-mode interferometer. This technology has been proven with an SU-8 core waveguide with an etched SU-8 nanograss scattering surface. This paper describes our creation of a fully silicon-based spectrometer using a silica core MMI waveguide. Scattering features were created in silica using SU-8 nanograss as an etch mask in a reactive ion etch (RIE). With optimized etch parameters, the silica core MMI spectrometer achieved an SNR of three with an incident light power of −68 dBm, which was almost 6 dB lower than designs with an SU-8 core.

## 1. Introduction

Optical spectrometers are essential tools in many diverse research fields, ranging from medicine to materials science [1,2]. Miniaturizing these devices by reducing their size and power consumption would increase their utility [3]. For example, miniature spectrometers in drones, spacecraft, or handheld devices could push productive science further and increase access to data. Many modern small-scale spectrometer systems suffer from poor wavelength resolution or a decreased effective wavelength range compared with larger tabletop devices. Increasing resolution and effective wavelength range while decreasing power requirements drives much of the research on miniaturized spectrometers [4]. 

Reconstructive spectrometers are a class of micro-spectrometers that use computational techniques to reconstruct spectral information from detector responses [4,5]. These devices have been categorized into two main groups: those that employ optical elements to produce a complex spatial light map representing a distinct spectrum, and those in which the light engages distinct spectral responses at an array of detectors [4]. Machine learning algorithms are trained on the device’s response to known spectra and then subsequently applied to interpreting responses produced from unknown spectra [5,6]. This technology has become viable on a micro-scale because of improvements to both fabrication techniques and computational power [4,7,8,9].

The multi-mode interferometer (MMI) spectrometer is one type of miniaturized reconstructive spectrometer that is effective in overcoming some of the challenges of miniature spectrometers [10]. MMI waveguides are photonic integrated circuit components that introduce light from one or more single-mode waveguides into a wider, multi-mode waveguide, which results in wavelength-dependent interference patterns [11,12]. As a photonic device, the MMI is typically used as a way to split light [11]. To use an MMI as a spectrometer, a top-down image of the wavelength-dependent modes is used in a machine learning algorithm to reconstruct the incident light spectrum [13].

Because light is typically confined inside the waveguide, artificially inducing light scattering on top of the waveguide can accentuate the modes inside the MMI to be imaged [14]. A visible light MMI spectrometer has been demonstrated for visible and near-infrared light using an SU-8 core [13]. SU-8, a common photodefinable polymer used in microfabrication [15], has the unique quality that when etched with an oxygen plasma, it develops nano-scale grass-like features. These features are effective for scattering visible wavelength light and allow for the internal modes of the MMI to be imaged [14]. These SU-8 MMI spectrometers have a signal-to-noise ratio (SNR) of 3 with an input power of −62.2 dBm and a mean square error (MSE) of 0.01 at inputs as low as 31.6 pW [10]. They also demonstrate 0.05 nm spectral resolution and have an effective wavelength range from the visible spectrum into the near-infrared region [10,13].

Here, we investigate the potential of creating an MMI spectrometer from a more durable, non-polymer material commonly used in microfabrication. Silicon dioxide (SiO_2_) was chosen as a candidate material because it is transparent to visible light and is a standard material in many microfabrication processes. Silica can be easily deposited at low temperatures using plasma-enhanced chemical vapor deposition (PECVD) [16]. Silica offers several advantages over SU-8, including a thermal expansion that would more closely match that of coupling devices and be effective over a larger temperature range. These qualities would enable the silica device to be better integrated with other microfabricated features and devices in a CMOS-compatible approach, as has been proven with a silicon nitride device [17]. This kind of fabrication will expand the impact of this device into the lab-on-chip field.

A necessary step in making silica MMI spectrometers was creating a light-scattering surface on the silicon dioxide comparable to the SU-8 nanograss. This paper details how SU-8 nanograss was etched into a thin layer of SU-8 over a silica MMI. The silica device was then etched using an anisotropic reactive ion etch (RIE). SU-8 grass over the oxide waveguide partially masked the silicon dioxide. This allowed for the transfer of nano features into the top of the silicon dioxide. Various etch lengths were tested in this study, and the resulting scattering coefficients from the roughened surfaces with visible light were measured.

## 2. Materials and Methods

### 2.1. Etching Silicon Dioxide for a Roughened Surface

**Figure 1:**
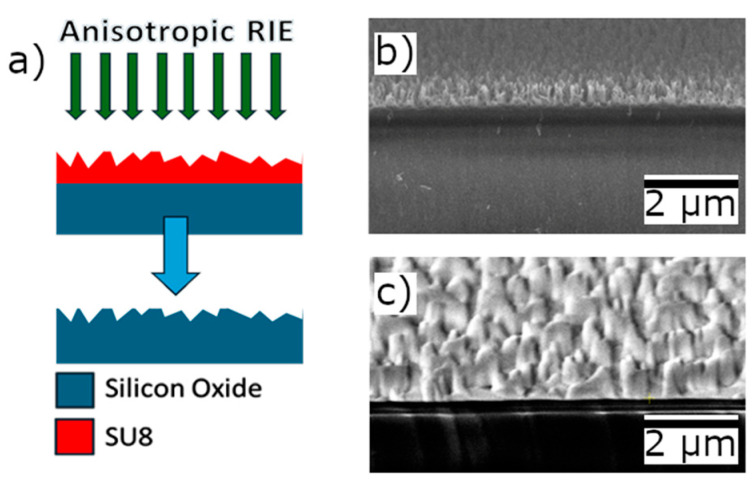
(**a**) Diagram depicting the process of using a roughened SU-8 mask to etch rough features into silica using an anisotropic RIE. The blue arrow shows the transfer of features into the substrate. (**b**) Electron microscope image of SU-8 nanograss on top of a silicon substrate. (**c**) Electron microscope image of silica nanofeatures etched in an anisotropic RIE with an SU-8 nanograss mask.

Fabricating roughened silicon dioxide using an anisotropic etch with a roughened SU-8 mask involved a modified version of a previously reported process [16]. The following steps are illustrated in Figure 1a: Silicon dioxide was grown on a silicon wafer using plasma-enhanced chemical vapor deposition (PECVD) in a PlasmaLab 80 Plus. SU-8 2005 was spin-coated onto the wafer at 3000 rpm for one minute. A series of thermal cycling and UV curing steps were then performed. The wafer was initially held at 65 °C for 8 min, then heated at 4 °C/min for 7.5 min to 95 °C and immediately cooled at 5 °C/min back to 65 °C. UV exposure was conducted using 365 nm light at 10 mW/cm^2^ for 55 s for curing. Following this, the wafer was reheated to 65 °C and held for 6 min. The temperature was subsequently increased at 5 °C/min for 6 min to a temperature of 95 °C, then cooled at 5 °C/min for 6 min back down to 65 °C. A final thermal cycle involved a 10 °C/ min ramp to 150 °C for 8.5 min, followed by cooling at 10 °C/ min back to 65 °C.

Following the full curing of the SU-8 layer deposited on PECVD silicon dioxide, the wafer was exposed to a 100 W oxygen plasma at a pressure of 75 mTorr for 22.2 min in an Anelva RIE DEM-451. Exposure of SU-8 to oxygen plasma results in the etching of grass-like structures, as shown in Figure 1b. These SU-8 nanograss structures are extremely fragile: they are susceptible to collapse and agglomeration if the wafer is exposed to liquid. Because of this, the wafer was transferred immediately to a CF4 reactive ion etch (RIE) in a Trion Technology Minilock Phantom III RIE/ICP. This etch was performed with a CF4 flow rate of 50 sccm at a pressure of 12 mTorr. The inductively coupled plasma (ICP) power was set to 275 W, and the reactive ion etch (RIE) power to 75 W. The etching duration was varied between 150, 200, 250, 300, 400, and 500 s.

The wafer was cleaned in Nanostrip at 90 °C for 10 min, in TC1 at 55 °C for 10 min, and then in oxygen plasma at 100 W for six minutes. The sample was etched in CF_4_ plasma at a pressure of 300 mTorr for 300 s. The resulting grass-like silica structures are shown in Figure 1c.

As can be seen in Figure 1b,c, the transfer of features from SU-8 to silica is not perfectly replicated. SU-8 nanograss is much finer, with widths of less than 100 nm. In contrast, silica nanofeatures are wider, at approximately 100 to 500 nm in width. This geometric discrepancy is attributable to the fragile nature of the SU-8 grass. It is hypothesized that the SU-8 nanograss clumps when exposed to CF4 plasma, leading to the larger features seen in the silica nanofeatures [16]. Despite these geometric differences, the resulting silica structures prove to be sufficiently rough to serve as an effective scattering surface. The heights of the SU-8 and silica nanofeatures are comparable. The comparable height is also likely due to SU-8 nanograss clumping.

### 2.2. MMI Spectrometer Fabrication and Testing

**Figure 2:**
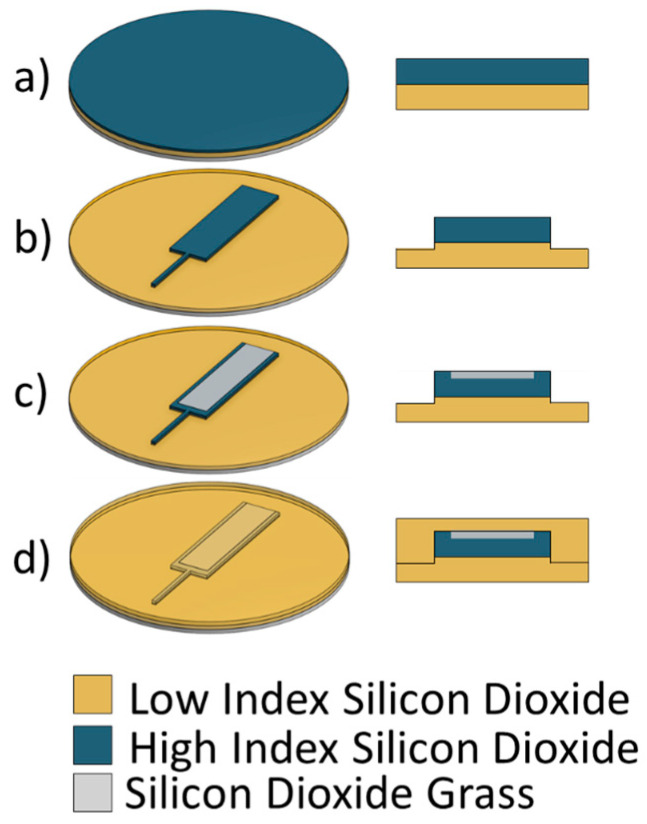
The fabrication process for creating a silicon dioxide MMI with a scattering region. (**a**) A high-index silicon dioxide thin film is grown using PECVD on top of a low-index PECVD silicon dioxide film. (**b**) MMI geometry is etched into the silicon dioxide. (**c**) A scattering region is etched into the MMI. (**d**) A low-index layer of PECVD silicon dioxide is deposited over the MMI as cladding.

To fabricate a silica MMI spectrometer with silica nanofeatures, a blank silicon wafer served as the substrate. On this silicon wafer, a 5 µm-thick layer of low-index (1.46) silicon dioxide was deposited using PECVD. This layer formed the lower cladding. On top of this layer, a silicon-rich, higher-index (1.51) silicon oxide layer was deposited, as shown in Figure 2a. This layer functioned as the waveguide core and was deposited to a thickness of 3 µm.

AZ3330 photoresist was patterned onto the wafer to define the waveguide core geometry. The wafer was cleaned by dipping it in hydrochloric acid, rinsing the acid off with deionized water, and then exposing it to 100 W oxygen plasma at 300 mTorr for one minute. Following cleaning, nickel was evaporated on the wafer, after which the AZ3330 was lifted off in acetone. The remaining nickel then served as an etch mask. This liftoff process was chosen because of the high edge quality that can be obtained compared with other methods. The wafer was etched in CF4 plasma using the same parameters described in the previous section. The wafer was etched for 1000 s to ensure the complete removal of all unprotected core-layer silicon dioxide. During this etching process, up to 1 µm of cladding silicon dioxide may have been removed. After the RIE step, the nickel mask was removed from the wafer using a nickel etchant.

The single-mode section of the waveguides, shown in Figure 2b, featured a rectangular cross-section measuring 3 µm in high and 4 µm in width. The single-mode waveguides were inserted into the center of the multi-mode waveguides. The multi-mode waveguides had dimensions of 3 µm in height and 100 µm in width. Both the single-mode and the multi-mode segments of the device collectively extended up to 2 cm long.

A scattering area on top of the waveguides started 2 mm from the point where the single-mode waveguide was inserted into the multi-mode waveguide. The width of the former was 70 µm across, and it was centered on the multi-mode waveguide so that there were 15 µm of non-scattering surface on either side. The length of the scattering area was 18 mm, running all the way to the end of the multi-mode waveguide, as shown in Figure 2c. The scattering areas on the waveguides were defined by photolithography with AZ3330. An aluminum mask was deposited on top of the wafer, and liftoff was done in acetone to expose the scattering regions. Silica grass was etched in the same manner described in the section above: SU-8 was spun onto the wafer, SU-8 grass was etched in oxygen plasma, and then, the silica was etched by a CF_4_ plasma. CF_4_ plasma etch times were varied to optimize light scattering. Different wafers were etched for 150, 200, 250, 300, 400 and 500 s.

After the grass section was etched, the aluminum mask was removed in aluminum etchant. The wafer was cleaned in nanostrip at 90 °C for 10 min, in TC1 at 55 °C for 10 min, and then in oxygen plasma at 100 W and a pressure of 300 mTorr for one minute. Water absorbed from the atmosphere into PECVD silica has been shown to locally change the index of refraction, altering the waveguiding properties [18]. To drive out the water and obtain a uniform refractive index through the core, wafers were annealed on a hotplate at 350 °C for 8 h. Low-index (1.46) PECVD SiO_2_ was deposited on top of the wafer to form the upper cladding, as shown in Figure 2d. The upper cladding layer protects the waveguide and the silica grass from being crushed or scratched and prevents water absorption.

**Figure 3:**
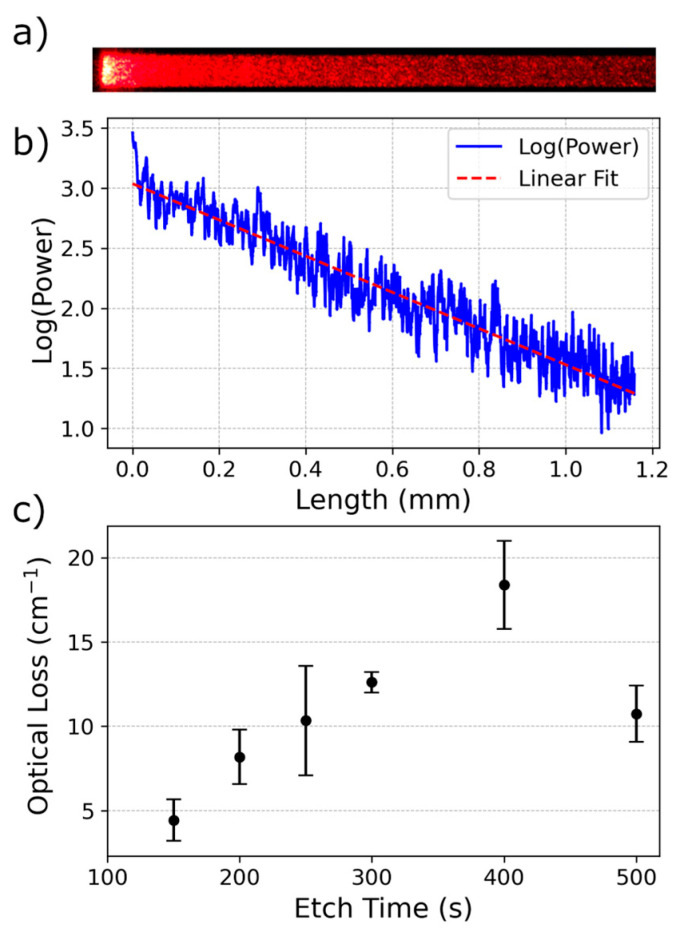
(**a**) Top-down image of the scattering region of the MMI with red laser light input. (**b**) Log of light emission power from a single MMI scattering region, plotted with length. A linear fit was calculated. (**c**) Mean with single standard deviation error bars for scattering coefficient at different etch times.

Chips with devices were cleaved from the wafer, and a camera was mounted above the device to observe light scattering from the roughened multi-mode section. A total of 0.02 mW of 635 nm laser light was butt-coupled into the waveguide from a fiber-optic cable. The camera was positioned to image the region of the scattering area closest to the single-mode waveguide. Images of the waveguides, like Figure 3a, were collected for analysis.

Scattering regions of the samples were etched by RIE for 150, 200, 250, 300, 400, or 500 s. Light emission from the scattering region of the waveguides was collected in the camera [13]. A one-dimensional representation of the waveguide was created by averaging 100 pixels from the middle of the waveguide across the entire length. Dark regions before the waveguide were excluded, as were the first 1000 pixels of the bright region of the waveguide. This was done to isolate the main light-scattering domain and exclude the higher scattering at the beginning of each waveguide. A linear fit was applied to the log of the data to find the loss coefficient. A representation of the fit for a 300 s etch waveguide can be seen in Figure 3b.

Numbers from the fits of at least 10 waveguides of each type were averaged to obtain a scattering value. This value can be seen in Figure 3c with error bars at one standard deviation. The trend appeared to be linear for etches of 150 through 400 s, and so a loss-to-etch-time relationship was calculated as

(1) $$Loss(t_{etch})=0.01803\;{cm}^{-1}s^{-1}\times t_{etch}-1.727\;{cm}^{-1}$$

It was assumed that the linear trend would continue, but the scattering surface etch began to remove so much of the MMI that light propagation through the waveguide was impacted, as seen with the 500 s etch sample.

Waveguides were then tested as a spectrometer by capturing two-dimensional data from images taken of the scattering region. These were fed into a neural network prediction model trained on each individual waveguide, as previously reported [13].

Our approach utilizes an end-to-end Convolutional Neural Network (CNN) for direct spectral reconstruction, which entails transferring non-linear mapping from the measured MMI scattering image to the target spectral output. The model’s architecture is centered on an initial Conv2D Layer designed to efficiently extract key spatial frequency and global interference patterns from the input image. These extracted features are then processed through a series of dense layers (the regression head) to map the visual information to the one-dimensional spectral intensity profile, which is carried out using a Sigmoid Activation Function on the output layer for intensity normalization. The network is trained using the Stochastic Gradient Descent (SGD) optimizer and the Mean Squared Error (MSE) loss function. Training stability is ensured through robust strategies like Early Stopping based on validation performance, which prevents overfitting and secures the best-performing model weights. The algorithm starts with a 120-by-1200-pixel image of the MMI scattering region that is passed through 512 20 × 20 convolution filters, which scan the image in 15 × 15 steps for overlap. This is followed by a rectified linear unit activation layer to generate a pool of features. These features range from local edges and gradients to the large-scale interference patterns. The feature maps are flattened and passed to a dense layer with 283,136 inputs and 200 outputs. Two additional dense layers of sizes 200→400 and 400→200 follow. The final layer produces the predicted spectral intensity vector.

## 3. Results and Discussion

**Figure 4:**
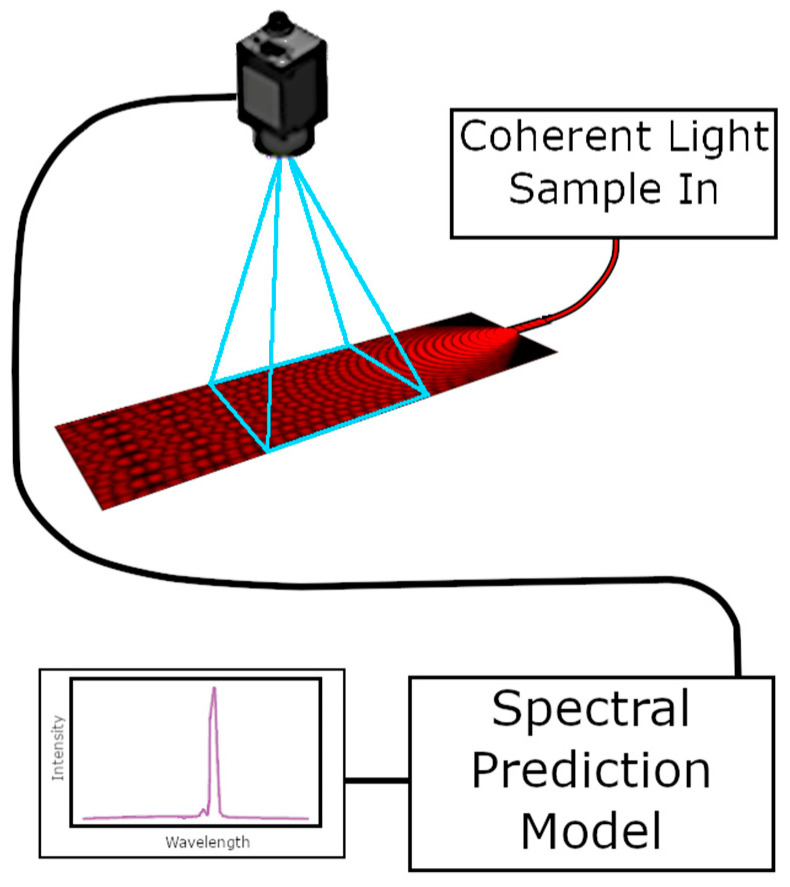
An image collected from the scattering region of the MMI is used as an input in a spectral prediction model that determines the wavelengths present.

**Figure 5:**
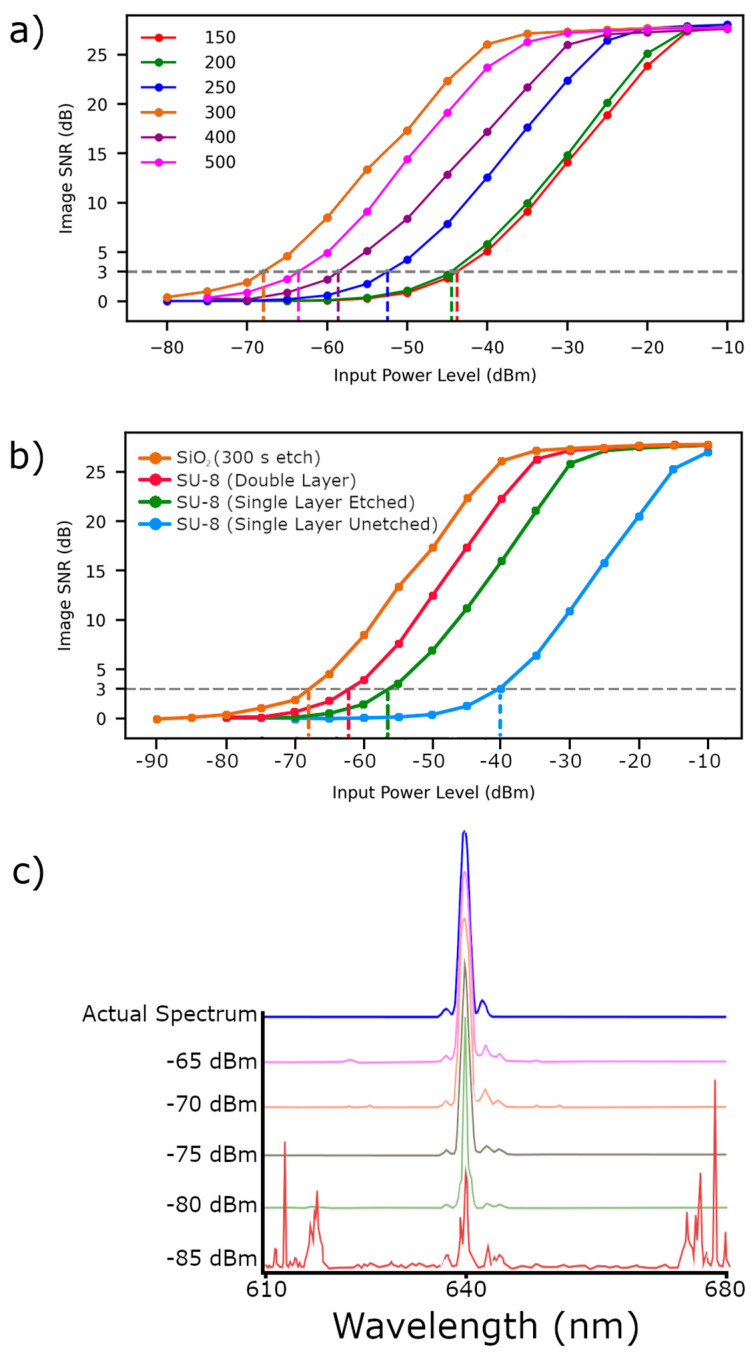
(**a**) Image SNR from silica MMI spectrometer devices with different etch times. (**b**) Image SNR from four different MMI spectrometer devices. The W300 silica device shows an improvement over the other three SU-8 devices. (**c**) Qualitative representation of spectrum prediction based on images from silica MMI spectrometer devices with light intensity ranging from −65 dBm to −85 dBm.

Grayscale images of the scattering region of the MMI were collected using an sCMOS sensor (Andor Zyla) with a 10× microscope objective, as shown in Figure 4. The image was cropped to a 1280 × 90 pixel region of interest (ROI) to balance computational capacity with information content. This image corresponded to an approximately 1280 × 90 µm^2^ region on the physical device. A CNN was trained to perform spectral prediction from these reduced images. Each device was trained separately. This mitigated the effect of individual variation between the devices. The supervised training used MMI scatter images generated with light from a supercontinuum laser filtered by a tunable Acousto-Optic Filter (AOTF) (NKT Photonics SuperK SELECT) to produce peaks between 620–671 nm in 1 nm steps (FWHM ≈ 1.6 nm). The corresponding spectra were simultaneously measured with a commercial optical spectrum analyzer (OSA) and used as training outputs. Training was conducted using an augmented dataset. This dataset was synthesized by constructing linear superpositions of the initial single-wavelength data pairs, thereby generating MMI images and spectral signatures corresponding to complex multi-peak spectra. This augmentation approach enabled the CNN to make robust predictions for both broadband and narrowband spectra [13]. This approach allowed for the effective identification of unknown spectra. The SNR of the image produced by the silica MMI spectrometers was measured. It was found that the 300 s etched device performed the best, with an SNR of 3 at a light input power of −68 dBm, as can be seen in Figure 5a.

The device with the highest scattering coefficient, the 400 s etch device, did not perform the best among the etched silica devices. Reconstructive spectroscopy relies on the creation of recognizable images corresponding to input wavelengths. When too much light scatters out, it may obscure hotspots that are indicative of wavelength. Alternatively, most of the light might emit at the beginning of the imaged region, not allowing for the formation of a longer, wavelength-dependent image. From our results, we conclude that the 300 s etch is optimal for creating the most sensitive MMI spectroscopy device.

Silica MMI spectrometers were compared with SU-8 core devices. The best of the silica MMI devices (300 s etched) improved in sensitivity over the best of the SU-8 core devices by 5.79 dBm. Results from these and the other SU-8 core devices can be seen in Figure 5b. Several graphs showing spectral predictions from the 300 s etch silica device at −65 to −85 dBm can be seen in Figure 5c. The spectrometer resolution was 0.05 nm. This value was limited by the training data and was not a physical limitation of the MMI spectrometer device. The dynamic range of the device ranges from UV (~400 nm) to near-IR (~1100 nm). This is based on the transparency of silica and the ability to establish distinguishable modes in the MMI spectrometer. As silica has a wider window of transparency than SU-8, particularly in the UV, a silica core MMI spectrometer should allow for a greater dynamic range than SU-8, though this has not been tested.

## 4. Conclusions

In this paper, we have detailed our fabrication and validation of a silica core MMI spectrometer. Fabricating the silica spectrometer required the transfer of light-scattering surface features similar to the nanograss etched on SU-8 MMI spectrometers into silica. This was done by using SU-8 nanograss as an etch mask over silica. An anisotropic reactive ion etch transfers similarly rough features into the silica. Different etch parameters were studied. A total of 400 s of etch time gave the highest scattering coefficient, but did not perform as well in terms of sensitivity when the device etched for 300 s. The optimized silica device showed a marked SNR improvement over the best SU-8 devices.

Though the scattering region of the devices fabricated for this study were 18 mm long, the active area imaged for spectrum reconstruction was approximately 1280 × 90 µm^2^. The scattering region started 2.5 mm after the beginning of the multi-mode waveguide. This made the necessary components of the device less than 4 mm long and 100 µm wide. An advantage of this narrow footprint and the fabrication process is that it would be relatively easy to have an array of many silica core MMI spectrometers. The Hamamatsu Model C14384MA is among the smallest commercially available chip-scale spectrometers. It has dimensions of 11.7 mm × 4.0 mm × 3.1 mm, with a spectral resolution of approximately 10 to 15 nm (FWHM) in the visible regime. The silica MMI spectrometer has an order of magnitude reduction in height and width while boasting a 0.05 nm spectral resolution in the visible regime.

The process of using SU-8 grass as an etch mask could have applications for other reconstructive spectrometers. Many reconstructive spectrometers rely on speckle patterns and machine learning algorithms for spectrum reconstruction [7,8,9]. This technique for roughening could enhance light emission for detection. Additional applications for roughened features using the SU-8 grass-masking technique include roughening of solar cells for increased efficiency as well as increasing surface area for catalysis [19,20].

## Data Availability

The original contributions presented in this study are included in the article. Further inquiries can be directed to the corresponding author.

## References

[1] MacRae M. (2019). Trends in Spectroscopy: A Snapshot of Notable Advances and Applications. Spectroscopy.

[2] Guan Q., Lim Z.H., Sun H., Chew J.X.Y., Zhou G. (2023). Review of Miniaturized Computational Spectrometers. Sensors.

[3] Bacon C.P., Mattley Y., DeFrece R. (2004). Miniature spectroscopic instrumentation: Applications to biology and chemistry. Rev. Sci. Instrum..

[4] Yang Z., Albrow-Owen T., Cai W., Hasan T. (2021). Miniaturization of optical spectrometers. Science.

[5] Zhang Y., Yang E., Yoon H.H., Cheng Q., Sun Z., Hasan T., Cai W. (2025). Reconstructive spectrometers: Hardware miniaturization and computational reconstruction. eLight.

[6] Zhang S., Dong Y., Fu H., Huang S.-L., Zhang L. (2018). A Spectral Reconstruction Algorithm of Miniature Spectrometer Based on Sparse Optimization and Dictionary Learning. Sensors.

[7] Xu Y., Wu J., Li H., Cai R., Zhu Y., Li Y., Shang T., Zhou H., Deng G. (2024). Compact speckle spectrometer using femtosecond laser-induced double-sided surface nanostructures. Opt. Lett..

[8] Zhou M., Kong H., Zhang Z., Li Y., Kang J., Yin L., Li Y., Wang L. (2025). A speckle enhanced prism spectrometer based on planar lightwave circuit chip. Opt. Commun..

[9] Feng F., Gan J., Chen P., Lin W., Chen G., Min C., Yuan X., Somekh M. (2022). AI-assisted spectrometer based on multi-mode optical fiber speckle patterns. Opt. Commun..

[10] Dixon P., Ramollari H., Carter A., Harkness J., Amin M.N., Pan D., Yuzvinsky T., Schmidt H., Hawkins A.R. (2025). Sub-Nanowatt Sensitivity MMI Spectrometers with Elevated Scattering Regions. J. Light. Technol..

[11] Cooney K., Peters F.H. (2016). Analysis of multimode interferometers. Opt. Express.

[12] Soldano L.B., Pennings E.C.M. (1995). Optical multi-mode interference devices based on self-imaging: Principles and applications. J. Light. Technol..

[13] Amin M.N., Ganjalizadeh V., Adams T.J., Dixon P.B., Weber Z., DeMartino M., Bundy K., Hawkins A.R., Schmidt H. (2024). Multi-mode interference waveguide chip-scale spectrometer (invited). APL Photonics.

[14] Adams T., Dixon P.B., Harkness J.G., Lee A.E., Amin M.N., Weber-Porter Z., Schmidt H., Hawkins A.R. Roughening of SU8 Waveguides to Accentuate Light Scattering. Proceedings of the 2024 Intermountain Engineering, Technology and Computing (IETC).

[15] Golvari P., Kuebler S.M. (2021). Fabrication of Functional Microdevices in SU-8 by Multi-Photon Lithography. Micromachines.

[16] Harkness J.G., Lee A.E., Dixon P.B., Yuzvinsky T.D., Schmidt H., Hawkins A.R. Nanoscale Surface Roughening Using SU-8 Nano Grass as an Etch Mask. Proceedings of the 2025 Intermountain Engineering, Technology and Computing (IETC).

[17] Ryckeboer E., Nie X., Subramanian A.Z., Martens D., Bienstman P., Clemmen S., Severi S., Jansen R., Roelkens G., Baets R. (2016). CMOS-compatible silicon nitride spectrometers for lab-on-a-chip spectral sensing. Silicon Photonics and Photonic Integrated Circuits V.

[18] Parks J.W., Cai H., Wall T., Stott M., Hamilton E., Chu R., Hawkins A.R., Schmidt H. Improvement of silicon dioxide ridge waveguides using low temperature thermal annealing. Proceedings of the 2015 Conference on Lasers and Electro-Optics (CLEO).

[19] Nunnari C., Fotia A., Malara A., Macario A., Frontera P. (2026). Micro- and Mesoporous Silica-Based Materials as Support Catalysts in Reforming Reactions. Catalysts.

[20] Trabelsi A.B.G., Velu Kaliyannan G., Gunasekaran R., Rathanasamy R., Palaniappan S.K., Alkallas F.H., Elsharkawy W.B., Mostafa A.M. (2024). Surface engineering of Sio_2_-Zro_2_ films for augmenting power conversion efficiency performance of silicon solar cells. J. Mater. Res. Technol..

